# Schizophrenia Etiological Factors and Their Correlation with the Imbalance of the Immune System: An Update -

**DOI:** 10.31661/gmj.v12i.3109

**Published:** 2023-12-01

**Authors:** Noushin Lotfi, Nahid Rezaei, Elham Rastgoo, Babak Khodadoustan Shahraki, Ghazaleh Zahedi, Morteza Jafarinia

**Affiliations:** ^1^ Department of Immunology, School of Medicine, Isfahan University of Medical Sciences, Isfahan, Iran; ^2^ Department of Immunology, School of Medicine, Lorestan University of Medical Sciences, Khorramabad, Iran; ^3^ Department of Radiology, School of Medicine, Shiraz University of Medical Sciences, Shiraz, Iran; ^4^ School of Medicine, Isfahan University of Medical Sciences, Isfahan, Iran; ^5^ Department of General Psychology, Iran University of Medical Sciences, Thran, Iran; ^6^ Shiraz Neuroscience Research Center, Shiraz University of Medical Sciences, Shiraz, Iran

**Keywords:** Schizophrenia, Immune System, Environmental Factors, Genes, Inflammation

## Abstract

Schizophrenia (SZ) is a severe psychiatric disorder associated with a dysregulation of the immune system. Immune-related genes and environmental factors including stress, food, infections, and microbiota, alter the immune system’s homeostasis and play a role in SZ pathogenesis. The most distinctive feature in the pathophysiology of the disease is a shift in the T helper 1(Th1)/Th2 balance toward Th2 dominance in the immune system. Also, microglial and Th17 cell activation cause inﬂammatory responses in the central nervous system (CNS). Antibodies play a role in the pathophysiology of SZ and give more evidence of a link between humoral immune reactivity and the disease. Accordingly, an imbalance in cytokine activities and neuroinflammation has been considered the main contributor to the pathogenesis of the SZ. Overall, the deregulation of the immune system caused by genetic, environmental, and neurochemical effects may all play a role in the etiology of SZ. This review summarized the etiological factors for SZ and discussed the role of immune responses and their interaction with genetic and environmental factors in SZ pathogenesis.

## Introduction

Schizophrenia (SZ) is a severe psychiatric disorder characterized by dysregulation of immune responses in the central nervous system (CNS). Although the etiology of SZ has not yet been determined, the impacts of environmental and genetic risk factors, including inflammatory-related genes, have been indicated in the disease [1][2][3]. Adverse immune responses in the CNS and a shift toward a T helper (Th) 2 immune response have been indicated in patients suffering from SZ [4]. Besides, the high prevalence of autoimmune diseases and the presence of autoantibodies against specific brain structures in حatient with SZ suggests that autoimmunity may play a role in the pathophysiology of the disease [5][6]. Also, prenatal exposure to infection has been suggested to increase the development of SZ in adolescents [7] and chronic inflammation in the CNS is established in patients with SZ [8]. Pro-inflammatory cytokines promote the degradation of tryptophan along the kynurenine pathway, which can lead to psychiatric abnormalities [9]. Analysis of neuroinflammation in postmortem brains of SZ subjects revealed increased microglial activity [10]. Infiltration of Th17 cells into the CNS is also involved in neuroinflammation in SZ [11]. This review will discuss the possible role of etiological factors, including the immune system, in SZ pathogenesis. 

1. Immune-related Genes in SZ

In recent years, methods including DNA/RNA chip technology have hardly been used in the assessment of neuroinflammation-related genes in SZ [12], so the association between inflammatory genes and SZ incidence has been identified [13]. 

Up-regulation of pro-inflammatory genes was observed in a patient with a recent onset of SZ. Several genetic studies on SZ have suggested that polymorphisms in the cluster of genes on human chromosome 6 (the major histocompatibility complex (MHC) region) are associated with SZ [14]. Also, the relation between inflammatory cytokine or chemokine genes and SZ has been indicated in previous studies. Association studies have suggested polymorphisms in pro-inflammatory cytokines, including genes coding for interleukin (IL)-6, tumor necrosis factor (TNF)-α, IL-1, and IL-8 genes, correlate with SZ outcome [15]. Polymorphism in these cytokines leads to the overproduction of pro-inflammatory cytokines without infection, usually found in SZ patients [14]. Furthermore, a missense mutation in neuregulin-1 (NRG-1) has increased cytokines including IL-8, TNF-α, and IL-6 in SZ subjects [16]. The association between cytokine gene polymorphisms with SZ has also been related to IL-2, IL-3, IL-3Rα, IL-4 genes, and the IL-10 promoter region [17][18].

2. Environmental Factors Affecting the Immune System in SZ

**Figure-1:**
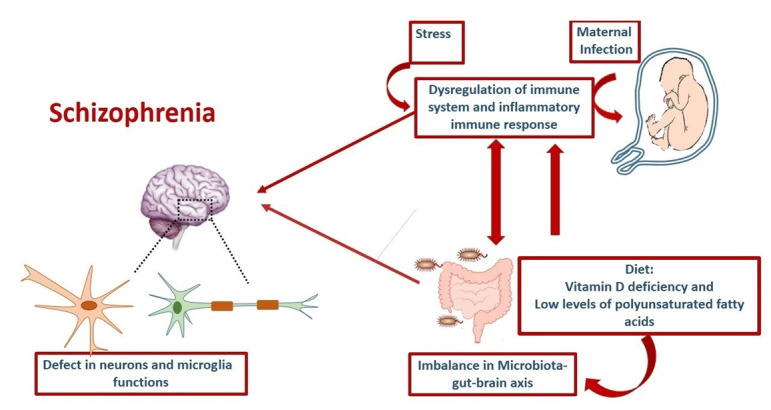
Figure 1- The impacts of environmental factors on the pathogenesis of schizophrenia. Environmental factors including maternal infections, diet, microbiota, and stress change the balance of the immune system and induce an inflammatory response that influences the CNS.**CNS:** Central Nervous System

Several environmental factors determine the pathobiology of SZ. Stress, diet, infections, urban living, and immigrant status are critical factors in SZ development [19]. These factors will be discussed in more detail (Figure-1).

2.1. Stress

Some evidence has shown that maternal stress during pregnancy may be associated with an increased risk of SZ in children [20]. Accordingly, a high rate of the disease has been reported in the children of women who were pregnant at the time of the German invasion of the Netherlands during World War II [21]. However, the effect of prenatal stress on the immune system and its role in SZ pathogenesis is controversial. Merlot et al. have suggested that prenatal stress leads to the downregulation of immune function [22]. A few studies have shown enhanced inflammatory immune responses after prenatal stress [23]. 

Some possible mechanisms for prenatal-stress-induced immune changes include; 1) the direct effect of maternal hormones and neurotransmitters on the ontogeny of immune cells, 2) deregulation of the hypothalamic-pituitary-adrenal (HPA)-axis in the prenatally stressed children, and 3) change in placental function via stress mediators [22]. Stress affects the HPA axis and sympathetic nervous system, leading to the production of neuroendocrine products including cortisol and catecholamines. These two primary mediators of stress can regulate various immune cell functions such as cytokine and chemokine production, trafficking, proliferation, and differentiation [24]. Studies have shown that stress has been associated with increased pro-inflammatory cytokines and reduced anti-inflammatory cytokine levels [25]. Animal model studies have also suggested that peripubertal stress can induce SZ-like behavioral changes (increased anxiety, reduced prepulse inhibition), neurotransmitter abnormalities (increased dopamine concentrations), and activation of hippocampal microglial cells [26]. Acute stress and early adverse life events may contribute to inflammation in patients with psychotic disorders, including SZ.

2.2. Diet

Diet is a second environmental factor that may be associated with psychiatric diseases, including SZ [27]. It is well-known that the immune system can be regulated by nutrient compounds including vitamin D, anti-oxidants, and polyunsaturated fatty acids (PUFA) [28]. Several studies have shown the low concentration of PUFA in both the brain and periphery of people with SZ. The decrease in PUFA may be due to the increased degradation of arachidonic acid. This state results from the altered immune function and overproduction of prostaglandins, especially prostaglandin E [29].

Regarding this view, recent studies have suggested that adjuvants including aspirin and celecoxib can lessen symptoms of SZ [30]. Different epidemiological studies have shown an inverse relation between fish consumption and mood disorders [31]. Certain omega-3 fatty acids are present in cell membranes, and a change in their concentrations could change the structure and function of various cell membrane proteins. Omega-6 fatty acids have the potential to cause inflammatory responses, but omega-3 fatty acids are recognized to have anti-inflammatory effects (Laye et al., 2018). So, an imbalance of omega-6/omega-3 could increase the production of pro-inflammatory cytokines [32][33] (Figure-1).

On the other hand, the association between prenatal exposure to famine and SZ susceptibility has been indicated in previous studies[34][35]. Accordingly, Xu et al. recently investigated the effect of starvation on SZ development in a rat model, and they found an essential role for famine-induced oxidative stress in SZ induction [36]. Additionally, oxidative stress disrupts the immune system’s balance, which may also contribute to the pathogenesis of SZ [37] (Figure-1).

2.3. Infection

Several environmental factors can elevate cytokine production during the prenatal period, and this overproduction affects neural development and psychiatric status [38][39]. Maternal infections are a significant candidate in SZ pathogenesis among the various environmental factors. Several studies established an association between maternal infection and an increased incidence of SZ in adulthood [40]. Moreover, the association between childhood infections in the CNS and SZ has been demonstrated [41][42][43]. Maternal infections could promote the release of cytokines and inflammation [44]. In this state, the inflammatory response can disrupt fetal brain development via increasing pro-inflammatory cytokines within the placenta, amniotic fluid, circulation, and brain [45]. Several studies of SZ have shown an increase in immune cells and pro-inflammatory cytokines during CNS infection [46]. They suggested that cytokines can disturb oligodendrocytes’ maturation and cause white matter abnormalities [21]. In line with this view, Brown et al. showed that elevated maternal level of the pro-inflammatory cytokine IL-8 due to infections in the second and third trimester of pregnancy was associated with an increased risk for SZ [47]. Another study also mentioned a relation between the blood levels of IL-8 in the mothers and structural brain abnormalities in schizophrenic children. [48] Maternal infections induce immune system activation, leading to the overproduction of pro-inflammatory cytokines in the placenta and amniotic fluid [48]. These cytokines may act on developing neurons and activate potential processes including astroglia and microglia stimulation (Figure-1). 

It has been documented that SZ is associated with several infectious agents, including cytomegalovirus [49], Toxoplasma gondii [50], influenza [51], measles [52], polio [53], herpes simplex virus type 2 (HSV-2) [54], diphtheria, and pneumonia [55]. Several studies have shown that the serum level of toxoplasma antibodies is higher in patients with SZ than in the general population. Also, mothers of children with SZ have higher IgG antibodies against toxoplasma [56]. 

Boska et al. reported that exposure of the developing fetus to influenza in the first trimester of pregnancy was accompanied by a seven-times higher chance of developing SZ [57]. Another study has shown that mothers who were seropositive for HSV-2 during pregnancy had twice the risk of their children developing SZ. Also, mothers exposed to rubella had a 10- to 20-fold increased risk of giving birth to a child with SZ. The effect of maternal infections on SZ development results from the immune responses, as most infections can’t cross the placenta (except parasitic infections, like toxoplasmosis). Likely, maternal antibodies cross the placenta and interact with fetal brain anti­gens, disrupting fetal brain development. However, some studies have also shown that many individuals exposed to similar infectious agents do not go on to develop SZ [58]. Recently, the role of microbiota in the balance of the immune system and neuronal development has been investigated. As a result, the microbiota-gut-brain axis regulates essential neural processes [59][60]. Xu et al. analyzed nineteen gut microbiota taxonomies in SZ subjects through two-stage metagenomic-wide association studies and calculated the index of microbial dysbiosis (MD). They found that the MD index was positively correlated with gut IgA levels but negatively associated with the gut microbiota population [61]. It seems that the gut microbiome contributed to the balance of the immune system and the SZ etiology. An imbalance of intestinal flora probably activates the immune system to produce inflammatory cytokines that disrupt synaptic/neuronal activity [62].

2.4. Urban Residence

Epidemiological studies have shown that the SZ prevalence rate increased with urban residence and higher population density [63]. This may be due to the higher prevalence of infectious diseases in urban areas [63]. Due to differences in socioeconomic status, culture, and access to medical health services, the distribution of patients with SZ was different in urban and rural areas [64]. Several underlying environmental factors in urban areas may contribute to disease pathogenesis [65].

2.5. Immigrant Status

Recent studies have shown that being a first- or second-generation immigrant is significant in how likely you are to get SZ. It may be due to psychosocial stress and the lack of innate immunity to a new environment, contributing to increased susceptibility to infection and potentially increased risk of SZ [66][67]. The prevalence of SZ is higher in developed or wealthier countries. It is probably because these countries are located at higher latitudes with more prenatal exposure to vitamin D deficiency and certain infections [68]. Vitamin D plays a role in brain development and immune function, and vitamin D deficiency may contribute to SZ by disrupting the early development of the nervous system. Maternal vitamin D deficiency may also increase therisk many of the prenatal infections pointed out in SZ [69].

3. Neurochemical Effects Mediated Immune System Imbalance 

Studying the metabolic pathway of the essential amino acid tryptophan could help shed light on the pathophysiology of SZ [70]. There are two metabolic pathways for breaking down tryptophan:

1. The methoxyindole pathway leads to the formation of the critical neurotransmitter 5-hydroxytryptamine or serotonin.

2. The kynurenine (KYN) pathway that induces the formation of kynurenic acid (KYNA) (an antagonist of the N-methyl-d-aspartate (NMDA) receptor) and quinolinic acid (QUIN) (NMDA receptor agonist) via the production of KYN [70].

Several studies have shown a change in KYN levels in SZ [71]. High levels of KYNA and its precursor KYN have been reported in CSF of patients with SZ [72]. Increased levels of KYNA induce SZ-like behavior such as disrupted prepulse in inhibition and auditory sensory gating, as well as impaired contextual discriminations, spatial working memory, and attentional set-shifting in rodents [73]. A study had shown that lower tryptophan and KYN could be measured in patients with SZ when they were added antipsychotic drugs and stimulated with LPS [74]. 

On the other side, meta-analysis studies have suggested that cytokine balance is shifted toward an inflammatory response in SZ [75][76]. Pro-inflammatory status leads to tryptophan metabolism more than the KYN arm [77][78]. Pro-inflammatory cytokines including interferon (IFN)-γ, TNF-α, IL-1β, or IL-6 are known to induce indolamine 2,3- dioxygenase (IDO) expression, the critical enzyme that degrades tryptophan to KYN and increases the level of KYN [79][80]. Stimulation of IDO enzymes results in the depletion of tryptophan and activation of kynurenine metabolites, as well as the release of neurotoxic glutamate [81]. In this state, a reduction in the serotonin secretion pathway leads to a decrease in serotonin synthesis. Serotonin decline is an essential determinant of the development of depression [80]. Together, the production of pro-inflammatory cytokines leads to a decrease in serotonin and a shift toward the formation of KYN, which has an apoptotic and neurotoxic effect [23]. 

4. Immune System Alternations in SZ

To better understand the impaired immune function in patients with SZ, we will provide a brief overview of innate and adaptive immune responses in SZ.

4.1. Innate Immune Responses 

Hyperactivation of the innate immune cells, including monocytes/macrophages in SZ, induces inflammation and the secretion of pro-inflammatory cytokines [82]. Cytokines cannot cross the blood-brain barrier (BBB) passively. Still, they might enter the brain under different conditions, such as increased permeability of the BBB with inflammation and stress or through circumventricular organs. Also, during inflammation, cytokines could be produced by resident immune cells of the brain, such as microglia [4]. The cytokines disrupt the important neurotransmitters and deregulate the neurodevelopmental systems, causing psychotic symptoms. They can also act directly on brain cells and activate IDO enzymes that result in the depletion of serotonin needed for tryptophan synthesis, triggering mood disorders [83]. Several studies have determined abnormal levels of pro-inflammatory cytokines and their receptors in the blood and CSF of schizophrenic patients and their relatives [84]. 

The roles of IL-6 and TNF-α are well understood in this field of study [85]. The high prevalence of IL-6 in SZ has been reported in many studies, but there are conflicting reports in this field. IL-6 increases the proliferation of B-lymphocytes and appears to play a crucial role in the immunologic abnormalities observed in patients with SZ. Also, IL-6 is associated with the deregulation of antibody production. So, the prevalence of autoantibodies may be due to increased cytokine concentrations. Elevated levels of IL-6 have been reported in mood disorders and possibly play a role in affective psychopathology in psychotic disorders [86].

Moreover, a significant increase in IL-6 production has been observed during acute stress [87]. It is worth mentioning that an elevated level of IL-6 is associated with more prolonged illness duration in patients with SZ [88]. Furthermore, high levels of IL-6 in SZ preserve more information on chronic immune activation and inflammatory syndromes in SZ [89]. IL-6 stimulates the release of acetylcholine, serotonin, and corticotropin-releasing hormone (CRH) in the CNS and plays an essential role in brain development, signal transduction, and behaviors related to feeding, sleep, and stress [90]. Alteration of plasma IL-6 level may be due to unspecific factors associated with the onset of SZ, including stress (social, travel, and infection) [89].

TNF-α and C reactive protein (CRP) have been found to rise in patients with SZ, which are important markers of innate immunity [91][92]. CRP assessment has been suggested as a potential agitation marker [91][93]. TNF-α plays an important role in neuroplasticity, cell resilience, and neuronal survival [94]. TNF-α levels are also elevated in this disease, indicating a genetic predisposition to the disease [95]. The TNF-α gene is found on the small arm of chromosome 6 near the MHC loci.

It has been expressed that this cytokine could be a susceptibility marker [96]. The high amounts of TNF-α promote Th1 and Th17 responses. They might be associated with SZ pathogenesis by activating the hypothalamic-pituitary-adrenocortical axis (HPA a-axis) and neuronal serotonin transporters. Therefore, IL-6 and TNF-α could be state or trait markers, respectively. Several studies have elucidated the complement proteins or genes’ dysregulation in patients with SZ, but their results are inconsistent. 

However, complement component 4 (C4), CUB (complement C1r/C1s, Uegf, Bmp1), and Sushi Multiple Domains 1 (CSMD1) have been recently introduced as potential genetic markers of SZ [97]. As an important index, the neutrophil-to-lymphocyte ratio (NLR) has also been examined in different studies [98]. In a meta-analysis study, the NLR was evaluated in subjects with SZ by Karageorgiou et al. They found an increase in NLR in both chronic disease and first-episode psychosis [99]. Microglia are resident macrophages in the brain and form approximately 15% of the CNS cells. They play different roles in immune response, neurodevelopment, and synaptic function. The role of microglia in the immune system as the first line of defense in the CNS is the secretion of inflammatory cytokines, free radicals, and anti-inflammatory components [100]. Microglia are divided into two subtypes, namely M1 and M2 microglia. M1 microglia are classically activated and have a pro-inflammatory function, while M2 cells are alternatively activated to express receptors and cytokines to inhibit inflammation and homeostasis [101]. 

There are various post-mortem and in vivo findings for microglia activation in SZ. In post-mortem studies, the activated microglia were identified by HLA-DR expression in a subset of schizophrenic patients [102]. Other studies showed an increase in HLA-DR+ microglia in the posterior hippocampus of paranoid schizophrenic patients who presented positive symptoms. This finding contrasts with patients who indicated negative symptoms and high CD3+ and CD20+ lymphocyte density in the same areas [103]. In SZ, there was a significant increase in HLA-DR+ microglial cell numbers in the frontal and temporal areas but not in the cingulate cortex. These cells had degenerative features leading to apoptotic changes [104]. The signs of activated microglia in the prefrontal and visual cortex have been observed. Microglia activity has also been followed by detecting calprotectin protein that binds to S100 protein.

Cell death occurs in this state due to high levels of exogenous calprotectin [105]. Activation of microglia in the body can be determined by PK11195, which is a ligand for benzodiazepine receptors, using the PET (positron emission tomography) technique [106]. The PET tracer binds to the mitochondrial translocator protein (TSPO) and leads to increases in activated microglia and pro-inflammatory astrocytes [107]. 

Two small studies have observed inflammation in the hippocampus and gray matter regions of schizophrenic patients compared to controls. This tracer could easily be used in human and animal model studies monitoring disease progression and therapy response in neurodegenerative patients. The findings have indicated that the activation of the immune system components, including microglia, occurring in the brain is associated with the specific illness process. This is supported by the fact that hippocampal functions like memory, sensory, and emotional are defective in patients with SZ [108].

**Figure-2:**
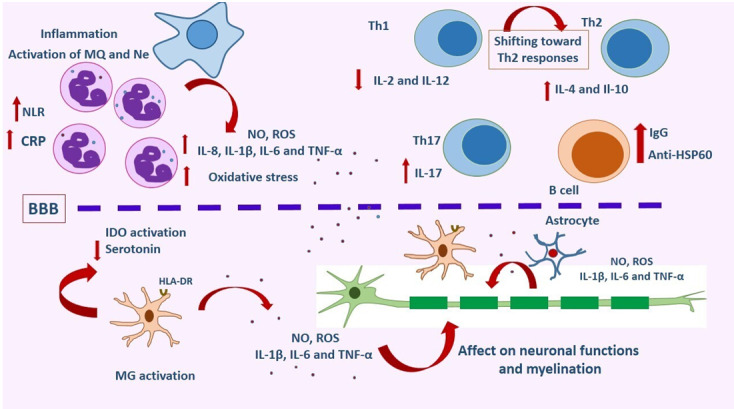
Figure 2- Immune system activation in schizophrenia. Innate immune cells such as MQ and Neutrophils are activated by environmental triggers. These cells produce pro-inflammatory cytokines such as IL-6, TNFα, IL-1, and IL-8. Activation of oxidative stress induces the production of NO and ROS. Also, CRP as an innate immune response protein and NLR are increased in patients with SZ. On the other side, activation of acquired immune cells such as Th2 and Th17, which produce IL-4, Il-10, and IL-17, plays an important role in SZ pathogenesis. While Th1 cytokines such as IL-12 and IL-12 are decreased in SZ. Activation of both innate and acquired immune cells influences BBB and induces inflammation in the CNS. In this state, microglia and astrocytes produce pro-inflammatory cytokines, NO, and ROS. Pro-inflammatory cytokines can also directly act on brain cells and activate IDO enzymes that result in the depletion of serotonin needed for tryptophan synthesis, which can trigger mood disorders. Numerous microglia cells show the expression of MHC II such as HLA-DR. **SZ:** schizophrenia, **MQ:** Macrophages, **Ne:** Neutrophil, **IL-6:** Interleukin-6, **IL-1:** Interleukin-1, **TNFα:** Tumor Necrosis Factor α, **IL-8:** Interleukin-8, **CRP:** C reactive protein, **NLR:** neutrophil-to-lymphocyte ratio, **Th1:** T helper1, **Th2:** T helper2, **Th17:** T helper17, **IL-2:** Interleukin-2, **IL-12:** Interleukin-12, **IL-10:** Interleukin-10, **IL-4:** Interleukin-4, **IL-17:** Interleukin-17, **BBB:** Blood-Brain Barrier, **NO:** Nitric Oxide, **ROS:** Reactive Oxigen Species, IDO: Indoleamine dioxygenase, MHC II: major histocompatibility complex class II.

Furthermore, other subjects are associated with microglial activation, including neurotransmitters. Activation of microglia could disrupt the regulation of neurotransmitters. The cytokines including IL-1β and IL-2 modulate the catecholamine concentration in the brain. The systemic application of IL-2 in patients undergoing anticancer therapy can result in psychotic or depressive symptoms. In addition to this, nitric oxide (NOS), produced from activated microglia, could influence cerebral monoaminergic substances and serotonin reduction. Additionally, reactive oxygen species (ROS) and cytokines produced by activated microglia can affect neuronal functions and myelination. Also, microglia are involved in the tryptophan degradation pathway, which produces quinolinic acid. Finally, increased concentrations of pro-inflammatory cytokines in the peripheral blood of subjects with SZ might lead to activation of the peripheral immune system and over-expression of these proteins. Besides disruption of the BBB, the increased cytokines can induce activation of microglia and astrocytes in the CNS [109] (Figure-2). 

4.2. Acquired Immune Responses

Studies have shown that Th1 cytokines including IL-2 and IL-12 are reduced in SZ while Th2 cytokines such as IL-10 and IL-4 are increased. IL-2 has a dose-related impact on dopamine secretion. High concentrations of IL-2 decrease dopamine, whereas low concentrations increase it. Many in vitro and in vivo studies confirmed the decreased production of IL-2 by lymphocytes, but some studies showed no change in IL-2 expression or even an increase in it [84]. One study demonstrated a correlation between low serum IL-2 and prolonged illness. In a recent report, increased serum levels of IL-2 were associated with increased severity of negative symptoms and tardive dyskinesia [15]. Also, several studies have shown that antipsychotic therapies are responsible for decreased IL-2 levels [110]. These findings suggest that IL-2 might have a crucial role in dopaminergic metabolism and psychotic symptoms in patients with SZ. 

Soluble intercellular adhesion molecule-1 (sICAM-1) is prominently expressed through Th1 activation and decreased levels in some patients with SZ, which may reflect the shifting of Th1 towards Th2-activity. Therefore, reduced levels of sICAM-1 may indicate reduced immune system activity, at least in a subgroup of patients patients with SZ. The presence of IgG and albumin in CSF is correlated with the progression of negative symptoms in SZ [111]. 

Also, antibodies against common antigens in the body, including heat-shock protein-60 (HSP-60) have been documented in SZ. They are a supportive sign of the theory that in some patients, Th2-activity is prominent [112]. HSP is involved in neuroprotective mechanisms, and antibodies against HSP inhibit neuroprotection [113].

Further, Riedel et al. have reported that skin responses to different antigens significantly decrease in patients suffering from SZ. These findings point to a clear attenuation of cellular immune response and support the theory of a shift toward Th2 [114]. However, other studies have reported different results regarding the immune system polarisation of Th2 cells. This hypothesis has been challenged and questioned by other studies and two recent meta-analyses. For example, the IL-4 level of the Th2 system does not increase at the onset of the first acute exacerbation. IL-10, another cytokine of Th2, has even been shown to decrease in patients with relapsed SZ and acute SZ [84]. IL-10 is an anti-inflammatory cytokine that plays a vital role in inhibiting pro-inflammatory cytokines [115]. 

Studies in animals or cell cultures have demonstrated the neuroprotective effect of IL-10 against glutamate-induced or hypoxic-ischaemic neuronal cell death, LPS- or interferon-induced oligodendrocyte cell death, and traumatic brain injury [116]. The mechanisms underlying IL-10’s neuroprotective effect on dopaminergic neurons are most likely due to the suppression of microglia or macrophage-mediated inflammatory mediator release. Because IL-10 has been reported to inhibit the production of cytokines such as TNF-α, IL-1β, IL-6, prostaglandin E2 (PGE2), ROS, and NO in glial cells [117]. Therefore, it seems that IL-10 may influence cognitive disorders in SZ through its neuroprotective action on dopaminergic neurons [118]. A decreased level of IL-10 has been reported in the early stages of SZ. Also, a low level of IL-10 has been associated with a high frequency of negative and cognitive symptoms in SZ [119]. In contrast, others have demonstrated unchanged levels in both medicated and unmedicated patients or even increased levels in SZ [119][120].

Th17 cells play an important role in autoimmunity, inflammation, and mucosal defense. These cells can secrete high concentrations of IL-17A, IL-17F, IL-21, IL-22, and GM-CSF. They infiltrate the CNS through disruption of the BBB by the direct effects of IL-17 and IL-22 [121]. Th17 cells are involved in the pathogenesis of encephalomyelitis and neuroinflammation in multiple sclerosis (MS) [122]. They activate microglia in the CNS and result in the production of IL1-β, TNF-α, and IL-6, which play a fundamental role in neuroinflammation. This process results from ROS production, which causes oxidative stress and might be used as a biomarker in the etiopathogenesis and follow-up of clinical periods in SZ [123]. Neuroinflammation has also been observed in several post-mortem brain studies showing upregulation of inflammatory genes [124]. Due to the importance of Th17 cells in the onset of encephalomyelitis and its correlation with SZ, Th17 activity can be imagined to play a role in SZ. Increasing Th17 cell activity has also been reported in bipolar disorder, autism, and depression [11]. 

Dopamine interacts directly with dopaminergic receptors on T cells and activates them through the upregulation of adhesion molecule expression. The increased expression of dopamine receptor D3 mRNA has been described in T cells of patients with SZ [125]. Also, stimulation of the dopamine D5 receptor, which is expressed on dendritic cells, could elicit the activation of Th17 cells. The antagonist of the D1 dopamine receptor can inhibit Th17 differentiation [126]. Attention to the hyperdopaminergic hypothesis in SZ suggests that Th17 cell responses could be enhanced by dopamine [127].

The role of the humoral immune system, as another arm of the specific immune response, has been indicated in the development of SZ. Hyperactivation of humoral immunity activates 2,3 IDO with the enhancing transformation of tryptophan to kynurenine, which acts as an NMDA antagonist [128]. However, some studies have emphasized the IDO enzyme catalyzation/degradation of L- tryptophan to KYN by Th1 cytokines. They again highlighted that oxidative stress can be associated with cellular immune activity (a Th1-like response) [29]. Many studies have reported increased levels of MCP-1 in patients with SZ. It seems that MCP-1 gene polymorphisms are responsible for resistance to antipsychotic therapy and can be found in patients suffering from severe subtypes of disease [129]. Enhanced levels of CCL11, which is a ligand for CCR3 and is expressed on Th2 cells, mast cells, and eosinophils, have been reported in SZ. This finding reinforces a shift toward increased Th2 response in SZ [130] (Figure-2).

5. Autoimmunity in SZ

Another immune-related theory in the SZ pathogenesis field is the autoimmune hypothesis. It was described by finding an increased rate of autoimmune diseases in relatives of patients with SZ, co-occurrence of autoimmune diseases and mental disorders, and common pathways between them [131]. Autoimmune diseases are characterized by failure to tolerate self-antigens, causing an immune response to these antigens, resulting in injury and tissue damage. For the development of autoimmune disorders, genetic susceptibility and a triggering event is usually needed, which may be either infection or another type of tissue injury [132]. Several autoimmune abnormalities have been associated with SZ [133]. SZ also has a high rate of co-morbidity with several autoimmune disorders, including Graves’s disease [134], multiple sclerosis [135], psoriasis [136], type-1 diabetes [137], and autoimmune hepatitis [138]. Based on a Danish study, a large population-based study, having an autoimmune disease increases SZ risk to 1.29. This risk is even higher in autoimmune hepatitis (2.75 fold), while rheumatoid arthritis is not associated with an increased risk of SZ [139]. In a study by Benros et al., it has been reported that autoimmune diseases increased the risk of SZ (1.29 fold), hospital-treated infections (1.60 fold), and 2.75 fold in the presence of both autoimmune disorders and hospital-treated infections [140]. These findings show that hospital-treated infections might trigger autoimmune disease and both conditions together (autoimmune disease and hospital-treated infections). These conditions highlight the fact that deregulation of the immune system can enhance the risk of SZ [141]. Some autoimmune disorders are caused by auto-antibodies reacting to systemic antigens, while others are specific to damaged organs.. Psychotic symptoms in mental disorders are associated with certain auto-antibodies, such as antibodies against the glutamate receptor and anti-p antibodies [139]. Antibodies against specific brain structures inc;uding the hippocampus, spectrum, cingulated gyrus, amygdala, and frontal cortex have also been recognized [142]. It has been suggested that reacting antibodies in the brain are evident and relevant in SZ [6]. In Benros’ study, autoimmune diseases in which brain antibodies are present have a higher association with SZ than other autoimmune disorders (1.48 vs. 1.19, respectively) [141]. 

Moreover, anti-cardiolipin, anti-nuclear, anti-DNA, and anti-histone antibodies have been reported in SZ. Other studies have indicated an association between antibodies against nerve growth factor (NGF), a neuro-specific protein, with positive symptoms and antibodies against leukocyte elastase (LE) with adverse symptoms in SZ [143]. Other antibodies including platelet-associated antibodies and muscarinic acetylcholine-receptor (mAChR) antibodies, particularly antibodies against the α-7 subunit of the acetylcholine nicotinic receptor (α7AChN-R, have been reported to have a positive relation with SZ [6]. The α7AChNR receptors modulate gamma-aminobutyric acid and glutamate neurotransmitter release, and reduced levels of those neurotransmitters are common findings in SZ [144]. According to Gallowitsch-Puerta and Tracey’s study, α 7AChNR also plays a role in balancing the pro-inflammatory and anti-inflammatory mechanisms of the immune system. It supports the theory of Th1-Th2 imbalance in SZ [145]. 

Many different antibodies may play a role in the development of SZ, but more research is needed to back up the findings made so far and show that the immune system isn’t working properly.

Autoimmune T-cell deficiency is another aspect of the immune disorder in SZ. This theory highlights the decreased response of T-cells (as evidenced by reduced production of cytokines) in some patients with SZ, which may result from neurodevelopmental damage following maternal immune activation [146]. Investigators have suggested that cytokine activity or inflammation in the fetal brain could disrupt the differentiation process of T cells and result in a reduced repertoire of naïve T cells [143]. Decreased T-cell function in SZ has also been demonstrated by reduced secretion of IL-2 from T-cells in untreated patients with SZ, have an attenuated response to antigens like myelin essential protein (MBP). Besides, impaired functional pathways of T-cells such as the cell cycle, oxidative stress, and intracellular signaling have been indicated [20]. It was suggested that this deficiency of T-cells could be associated with the onset or progress of SZ. This theory is further supported by the observation in mouse models of disturbed homeostasis of neurotransmitter production, including glutamine and dopamine. Thus, psychotic behavior could potentially be improved by increasing the autoimmune T-cell population [147].

## Conclusion

Alterations of the immune system and inflammation might contribute to pathological processes in SZ. In addition, immune-related genes and environmental factors, including stress, food, infections, and microbiota, alter the immune system’s homeostasis and play a role in SZ pathogenesis. Neuroinflammation has been suggested as the primary cause of SZ pathogenesis.

According to previous studies, the genes involved in SZ are predominantly inflammatory. The most distinguishing hallmark of the disease pathophysiology is a shift in the Th1/Th2 balance toward Th2 dominance in the immune system. Furthermore, the link between serum level antibodies and the progression of negative symptoms in SZ is a piece of positive evidence for the theory that Th2-activity is prominent in some individuals. Antibodies play a part in the pathophysiology of SZ and give more evidence of a link between humoral immune reactivity and the disease. It should also be noted that T-cell function is significantly decreased, and there is no obvious cellular immune reaction in patients with SZ. SZ appears to be a disease with different pathological processes. Overall, the deregulation of the immune system caused by genetic, environmental, and neurochemical effects may all play a role in the etiology of SZ.

## Conflict of Interest

None.
